# Association between heart rate recovery after exercise and renal function in patients referred for treadmill exercise test

**DOI:** 10.1371/journal.pone.0222236

**Published:** 2019-09-06

**Authors:** Rei-Yeuh Chang, Han-Lin Tsai, Ping-Gune Hsiao, Chao-Wen Tan, Chi-Pin Lee, I-Tseng Chu, Yung-Ping Chen, Malcolm Koo

**Affiliations:** 1 Division of Cardiology, Department of Internal Medicine, Ditmanson Medical Foundation Chia-Yi Christian Hospital, Chiayi City, Taiwan; 2 Chung Jen Junior College of Nursing, Health Sciences and Management, Chiayi, Taiwan; 3 Min-Hwei Junior College of Health Care Management, Tainan City, Taiwan; 4 Graduate Institute of Long-term Care, Tzu Chi University of Science and Technology, Hualien City, Hualien, Taiwan; 5 Dalla Lana School of Public Health, University of Toronto, Toronto, Ontario, Canada; West Virginia University, UNITED STATES

## Abstract

**Introduction:**

Heart rate recovery (HRR) is a marker of parasympathetic activity recovery after exercise, and it is associated with cardiovascular mortality and total mortality. Impaired renal function is also associated with cardiac mortality. The aim of this study was to investigate the association between HRR after exercise and renal function in patients referred for a treadmill exercise test.

**Patients and methods:**

This cross-sectional study was conducted at a regional hospital in southern Taiwan. Patients who completed a symptom-limited treadmill exercise test from January 2015 to February 2018 were recruited. Before the treadmill exercise test, patients were asked to complete a questionnaire on the past disease history and lifestyle factors. Serum creatinine measurement within two years prior to or after the date of the treadmill exercise test of the patients was also obtained from the medical records for these patients. Estimated glomerular filtration rate (eGFR) was calculated. Simple and multiple linear regression analyses were performed to investigate the association between one-minute HRR and eGFR.

**Results:**

A total of 2,825 patients completed the treadmill exercise test, and serum creatinine measurement was identified from medical records for 2,153 patients (76.2%). Multiple linear regression analysis revealed that a lower eGFR was significantly associated with lower one-minute HRR (*P*< 0.001), adjusting for other significant independent factors, including age, waist circumference, type 2 diabetes mellitus, and smoking.

**Conclusions:**

In this cross-sectional observational study, a lower eGFR was significantly and independently associated with decreased one-minute HRR, suggesting that parasympathetic activity recovery after exercise could be impaired by a decrease in renal function.

## Introduction

The treadmill exercise test is a widely used tool to diagnose obstructive coronary artery disease. Hemodynamic data during treadmill exercise test can also provide valuable diagnostic information, including exercise capacity, heart rate reserve, blood pressure reserve, rate-pressure product, chronotropic reserve, heart rate recovery, rate-pressure product reserve, and arrhythmia. Heart rate recovery (HRR) is a decrease in heart rate immediately after termination of exercise, which is related to the restoration of the parasympathetic nervous system after exercise [1]. Older age, low physical activity [2], metabolic syndrome [3], type 2 diabetes mellitus [4, 5], and smoking [6] had been reported to be associated with a low HRR.

Previous studies indicated that a low HRR was independently associated with cardiovascular mortality and all-cause mortality [7, 8]. A low HRR was also related to an increased risk of sudden death from myocardial infarction, but not non-sudden death from myocardial infarction [9]. Vagal tone restoration post exercise appeared to play a critical role in the prevention of potentially lethal arrhythmias and sudden death. An attenuated HRR was also associated with advanced coronary artery calcification independent of traditional coronary artery disease risk factors and the development of new-onset atrial fibrillation [10, 11].

Chronic kidney disease (CKD) is condition characterized by a gradual and progressive loss of kidney function over time, and is defined as a reduced glomerular filtration rate (GFR), increased urinary albumin excretion, or both, present for over 3 months. Globally, the burden of CKD continues to increase and the prevalence is estimated to be 8 to 16% [12]. CKD was first defined based on the National Kidney Foundation (NKF) Kidney Disease Outcomes Quality Initiative (KDOQI) introduced in 2002 [13] and endorsed at subsequent Kidney Disease Improving Global Outcomes (KDIGO) Controversies Conferences with minor modifications in 2004 and further in 2018 [14, 15]. The classification is widely used in clinical practice, research, prevention, and treatment of CKD. A large community-based study showed that a low estimated glomerular filtration rate (eGFR) was associated with increased risk for cardiovascular events, hospitalization, and mortality [16–18]. In addition, a recent meta-analysis of 83 studies reporting 30,392 strokes revealed that the risk of stroke increased linearly and additively with declining eGFR [19].

Although both a low HRR and eGFR have been reported to be associated with an increased cardiovascular risk, few studies have investigated the association between HRR and eGFR. A study on 107 patients with CKD had directly investigated the association between HRR and eGFR. Results from multiple linear regression analysis indicated that reduced eGFR was a significant and independent risk factor for a lower HRR [20]. Therefore, the aim of this cross-sectional study with a large sample size was to evaluate the association between eGFR and HRR in Taiwanese patients referred for a treadmill exercise test.

## Patients and methods

### Study design and study population

A cross-sectional observational study design was used. The study participants included all patients referred for treadmill exercise test at a regional hospital in southern Taiwan between January 2015 and February 2018. Patients were excluded if they had an implanted pacemaker or atrial fibrillation. The study protocol was approved by the Institutional Review Board of Chia-Yi Christian Hospital, Taiwan (CYCH-IRB No. 104008). All patients provided written informed consent.

### Treadmill exercise testing

After a supine and standing electrocardiography (ECG), heart rate, and blood pressure were obtained, a symptom-limited treadmill exercise testing using the standard Bruce protocol was performed (GE T-2000 treadmill, CardioSoft diagnostic software version 5.20, Marquette ECG analysis program, GE Medical Systems IT, Inc. Milwaukee, USA). Blood pressure was measured non-invasively using a Suntech 4240 monitor (Suntech Medical Instruments, Raleigh, NC) from left brachial artery. Patients were encouraged to achieve a goal of 85% of maximum age-predicted heart rate (HRmax) (beats/min), which was calculated by 220 minus the age of the participants. Percentage of maximum age predicted heart rate (%HRmax) was calculated by (peak heart rate/maximum age predicted heart rate) × 100. Functional capacity was estimated based on a range of speeds and grades of the treadmill. It was expressed in metabolic equivalent of task (MET), where one MET is equivalent to 3.5 mL/kg/min of oxygen consumption. ECG, heart rate, and blood pressure were recorded at each stage of exercise, peak exercise, and every minute in the recovery phase up to 6 minutes. A positive ischemic ST-segment response was defined as the horizontal or downsloping ST-segment depression of > 1 mm below baseline taken 80 ms after the J-point. Rate-pressure product (RPP) (mmHg × bpm × 10^−2^) was calculated as the product of systolic blood pressure (mmHg) and heart rate (beats/min) at peak exercise and then divided by 100. The dependent variable in this study was one-minute HRR, which was defined as the decrease in heart rate from peak exercise to 1 minute after exercise. All treadmill exercise testing data were obtained from chart review.

### Clinical data and questionnaire

A medical chart review was conducted to obtain measurements on serum creatinine of the patients who had completed the treadmill exercise test. Only patients with serum creatinine measurement within two years prior to or after the date of the treadmill exercise test were included in the study. eGFR was calculated with the Modification of Diet in Renal Disease (MDRD) equation [eGFR (mL/min/1.73m^2^) = 186 × serum creatinine ^-1.154^ × age^-0.203^ (× 0.742 if female)] [21]. In addition, the values of eGFR≥ 90 mL/min/1.73 m^2^ are defined as CKD stage 1 or normal. The values of eGFR between 60‒89 mL/min/1.73 m^2^ are defined as CKD stage 2. The values of eGFR between 45‒59 mL/min/1.73 m^2^ are defined as CKD stage 3a. The values of eGFR between 30‒44 mL/min/1.73 m^2^ are defined as CKD stage 3b. The values of eGFR between 15‒29 mL/min/1.73 m^2^ are defined as CKD stage 4. The values of eGFR < 15 mL/min/1.73 m^2^ are defined as CKD stage 5.

Body weight, body height, and waist circumference of the patients were measured prior to the treadmill exercise test. Moreover, the patients were asked to complete a questionnaire on past history of diseases (including hypertension, diabetes mellitus, coronary artery disease, hyperlipidemia, chronic obstructive pulmonary disease, and renal disease), lifestyle factors (smoking, drinking, exercise, and perceived health status), and sleep quality. The Pittsburgh Sleep Quality Index (PSQI) questionnaire was used to assess sleep quality over the previous one-month period. A PSQI global score was calculated with a range from 0 to 21 points. A score of > 5 is indicative of poor sleep quality [22].

### Statistical analysis

Continuous variables are expressed as mean and standard deviation (SD). Categorical data are expressed as number and percentage. Simple linear regression and stepwise multiple linear regression analyses were conducted to investigate the association between HRR and renal function. Two multiple linear regression models were evaluated with renal function expressed as either stages of CKD (Model 1) or eGFR (Model 2). A stepwise regression method was used to obtain the final model, adjusting for potential confounding variables. All statistical analyses were performed with PASW Statistics for Windows, version 18.0 (IBM Corp., Chicago, USA). A two-tailed *P* value of < 0.05 was considered statistically significant.

## Results

Of the 2,825 patients completed the treadmill exercise test, serum creatinine measurement could be identified for 2,153 patients (76.2%) from the medical records. The mean duration between the treadmill exercise test and creatinine measurement was 20.4 days (SD 159.8 days) with 40% of the duration were within 30 days. The basic demographic and clinical data of the patients are shown in Table 1. The mean age was 53.4 years and 55.6% were male patients. The mean body mass index was 25.6 kg/m^2^ and the mean waist circumference was 86.3 cm. The mean eGFR was 106.6 mL/min/1.73m^2^. Moreover, 72.7% of the patients were CKD stage 1, 23.2% were stage 2, 2.6% were stage 3a, 0.7% were stage 3b, 0.3% were stage 4, and 0.6% were stage 5.

**Table 1:** Demographic and clinical characteristics of study participants (N = 2153).

Variable	Number (%) or mean (standard deviation)
Age, years	53.4 (11.7)
Sex, male	1196 (55.6)
Body mass index, kg/m^2^	25.6 (3.9)
Waist circumference, cm	86.3 (10.6)
eGFR, mL/min/1.73m^2^	106.6 (29.8)
Chronic kidney disease stage	
Stage 1	1566 (72.7)
Stage 2	499 (23.2)
Stage 3a	56 (2.6)
Stage 3b	14 (0.7)
Stage 4	6 (0.3)
Stage 5	12 (0.6)
Hypertension	786 (36.5)
Type 2 diabetes mellitus	324 (15.0)
Coronary artery disease	327 (15.2)
Hyperlipidemia	406 (18.9)
Chronic obstructive pulmonary disease	26 (1.2)
Smoking	380 (17.6)
Alcohol use	363 (16.9)
Exercise	1296 (60.2)
Poor sleep quality (PSQI > 5)	233 (10.8)
Perceived health status	
Very good or good	232 (10.8)
Fair	1275 (59.2)
Poor or very poor	646 (30.0)

eGFR, estimated glomerular filtration rate; PSQI, Pittsburgh Sleep Quality Index.

Chronic kidney disease stages were defined as follows: stage 1, eGFR≥ 90 mL/min/1.73m^2^; stage 2, eGFR 60–89 mL/min/1.73m^2^; stage 3a, eGFR 45–59 mL/min/1.73m^2^; stage 3b, eGFR 30–44 mL/min/1.73m^2^; stage 4, eGFR 15–29 mL/min/1.73m^2^; and stage 5, eGFR<15 mL/min/1.73m^2^.

Poor sleep quality was defined as a Pittsburgh Sleep Quality Index score of > 5.

Exercise was dichotomized with a cut-off of ≥ 3 days/week with duration ≥ 30 min.

Treadmill exercise test results showed that the mean one-minute HRR was 22.1 beats/min (SD 9.5), RPP reserve was 16.2 mmHg × bpm × 10^−2^ (SD 4.6), functional capacity was 9.5 METs (SD 2.9), ventricular premature contractions (VPCs) was 11.9%, atrial premature contractions (APCs) was 5.2%, and ischemic ST-T change during exercise was 19.7% (Table 2).

**Table 2:** Results of treadmill maximal exercise test.

Variable	Number (%) or mean (standard deviation)
One-minute heart rate recovery, bpm	22.1 (9.5)
Rate-pressure product reserve, mmHg × bpm × 10^−2^	16.2 (4.6)
Functional capacity, MET	9.5 (2.9)
Arrhythmia	
APC	112 (5.2)
VPC	256 (11.9)
SVT	6 (0.3)
AF	4 (0.2)
VT	4 (0.2)
Ischemia	
Negative	1598 (74.2)
Positive	424 (19.7)
Inconclusive	131 (6.1)

AF, atrial flutter; APC, atrial premature contraction; BPM, beats per minute; MET, metabolic equivalent of task; SVT, supraventricular tachycardia; VPC, ventricular premature contraction; VT, ventricular tachycardia.

Table 3 shows the results of simple linear regression analysis, and two models of multiple linear regression analyses, using either stages of CKD (Model 1) or eGFR (Model 2) as a measure of renal function. Simple linear regression analysis revealed that age, sex, body mass index, waist circumference, hypertension, type 2 diabetes mellitus, coronary artery disease, hyperlipidemia, smoking, poor or very poor perceived health status, eGFR, and stages of CKD were significantly associated with one-minute HRR. In addition, multiple linear regression analyses showed that renal function expressed by either CKD stage (stage 2, stage 3a, and stage 3b, 4, 5) or eGFR was significantly and independently associated with one-minute HRR, adjusting for age, waist circumference, type 2 diabetes mellitus, and smoking. The regression coefficients associated with stage 2, stage 3a, and stage 3b, 4, 5 were −1.94, −3.96, and −5.20, respectively, with a dose-response pattern (*P* for trend < 0.001). The regression coefficient for eGFR was 0.036 (*P* < 0.001).

**Table 3:** Simple and multiple linear regression analyses of one-minute heart rate recovery.

Variable	Simple linear regression			Multiple linear regression Model 1			Multiple linear regression Model 2		
	b	Std β	*P*	b	Std β	*P*	b	Std β	*P*
Age, years	−0.175	−0.216	< 0.001	−0.145	−0.179	< 0.001	−0.137	−0.169	< 0.001
Sex (female as reference)	−2.053	−0.107	< 0.001	–	–	–	–	–	–
Body mass index, kg/m^2^	−0.392	−0.160	< 0.001	–	–	–	–	–	–
Waist circumference, cm	−0.182	−0.203	< 0.001	−0.138	−0.154	< 0.001	−0.139	−0.155	< 0.001
Hypertension	−2.668	−0.135	< 0.001	–	–	–	–	–	–
Type 2 diabetes mellitus	−4.662	−0.175	< 0.001	−2.338	−0.088	< 0.001	−2.702	−0.101	< 0.001
Coronary artery disease	−2.081	−0.078	< 0.001	–	–	–	–	–	–
Hyperlipidemia	−1.297	−0.053	0.014	–	–	–	–	–	–
Chronic obstructive pulmonary disease	−1.869	−0.021	0.321	–	–	–	–	–	–
Smoking	−1.905	−0.076	< 0.001	−2.057	−0.082	< 0.001	−1.949	−0.078	< 0.001
Alcohol use	−0.749	−0.029	0.173	–	–	–	–	–	–
Exercise	0.169	0.009	0.687	–	–	–	–	–	–
Perceived health status (very good or good as reference)				–			–	–	–
Fair	−0.636	−0.033	0.351	–	–	–	–	–	–
Poor or very poor	−1.487	−0.071	0.042	–	–	–	–	–	–
Poor sleep quality	−0.891	−0.029	0.179	–	–	–	–	–	–
Estimated glomerular filtration rate, mL/min/1.73m^2^	0.065	0.202	< 0.001	Not evaluated in Model 1			0.036	0.112	< 0.001
Chronic kidney disease stage (stage 1 as reference)							Not evaluated in Model 2		
Stage 2	−3.574	−0.155	< 0.001	−1.940	−0.084	< 0.001	–	–	–
Stage 3a	−7.708	−0.118	< 0.001	−3.958	−0.061	0.004	–	–	–
Stages 3b, 4, 5	−8.169	−0.100	< 0.001	−5.197	−0.064	0.002	–	–	–

b: beta coefficient; std β: standardized beta coefficient.

Exercise was dichotomized with a cut-off of ≥ 3 days/week with duration ≥ 30 min.

Poor sleep quality was defined as a Pittsburgh Sleep Quality Index score of > 5.

Linear trend test for the stages of chronic kidney disease, *P* < 0.001.

## Discussion

This cross-sectional study revealed that renal function, expressed as either CKD stage (stage 2, stage 3a, and stage 3b, 4, 5) or eGFR was significantly and independently associated with one-minute HRR in adults. Késoi et al. reported a significant and independent association between eGFR and HRR in Hungarian patients with biopsy-confirmed chronic IgA nephropathy [20]. A slower one-minute HRR after exercise stress test was also observed in patients with primary nephrotic syndrome [23]. Our findings generalize these results to the Taiwanese population with a larger sample size of 2,153 patients. We also showed that a significant association between impaired renal function and HRR could be observed regardless of whether renal function was expressed either as a continuous variable eGFR or when it was categorized as stages of CKD.

The correlation between renal function and HRR could be related to autonomic dysfunction in patients with CKD [24]. Several possible mechanisms have been proposed to the dysfunction, including impaired reflex control of autonomic activity, activation of the renin-angiotensin-aldosterone system, activation of renal afferents, structural remodeling of the heart and vasculature, decreased nitric oxide bioavailability, and increased mental stress. Nevertheless, the precise mechanism is still unclear [25].

In addition to CKD stages, an older age, a larger waist circumference, type 2 diabetes mellitus, and smoking were also found to be independently associated with a low one-minute HRR in the present study. Sympathetic responses and parasympathetic responses are known to decline with increasing age [26]. The Coronary Artery Risk Development in Young Adults (CARDIA) cohort study showed that HRR declined 2.5 beats/min over 7 years. The study also found that participants with increased physical activity during the study period had the least declined HRR (−1.3 beats/min), whereas those with decreased physical activity had the most declined HRR (−3.6 beats/min) [2]. Many studies aimed to modify HRR by exercise training or rehabilitation program. For example, an exercise training study revealed that high intensity interval training could improve HRR, but there was no improvement after a high volume of low intensity training [27]. Another cardiac rehabilitation study revealed that a cardiac rehabilitation exercise program had a positive effect on HRR, while home-based exercise group was not able to improve HRR [28]. In addition, a study on patients with stable chronic heart failure reported that continuous but not interval exercise training could improve HRR [29]. In the present study, exercise ≥ 3 days/week with a duration ≥ 30 min was not significantly associated with HRR. Since exercise intensity was not well defined by our questionnaire, further study on the association between HRR and exercise should include measurement of intensity in addition to duration and frequency. Further studies using a longitudinal design should be conducted in patients with CKD to clarify whether the restoration of parasympathetic tone by increasing HRR could decrease the progression of CKD.

Waist circumference was found to be significantly associated with one-minute HRR in the present study. Waist circumference, but not body mass index has previously been shown to inversely associate with HRR in obese individuals [3]. Visceral abdominal fat was found to be associated with autonomic function on HRV assessment [30], which might be related to catecholamine production and adipocytokines secretion from fat cells [31, 32]. A study from the National Health and Nutrition Examination Survey in Taiwan also revealed that waist circumference, systolic blood pressure, serum glucose, serum C-reactive protein were inversely associated with HRR in girls, but only waist circumference associated was associated with HRR in boys [33].

In our study, type 2 diabetes was inversely associated with one-minute HRR, indicating that patients with diabetes had declined parasympathetic recovery post exercise. A cohort study of 1,818 patients underwent a routine coronary artery disease screening program also found an increased risk of abnormal HRR in patients with diabetes, even after adjusting for a number of potential confounding factors [4]. A recent study on 123 patients also reported that post-exercise cardiac autonomic recovery as assessed by HRR and heart rate variability was found to be impaired in patients with type 2 diabetes, and the effects were more pronounced in patients with poor glycemic control [34]. Another study aimed to investigate the risk factors of HRR in patients with type 2 diabetes reported that fasting blood glucose, glycosylated hemoglobin, low-density lipoprotein cholesterol, and resting and maximum heart rates were significantly associated with HRR. The study also found that a combined aerobic and resistance training program of moderate intensity was able to improve HRR, possibly due to better glycemic control, resting heart rate, and physical fitness [35].

Smoking was found to be associated with a lower one-minute HRR in the present study. Smoking is one of the major cardiovascular risk factors and its pathophysiology includes platelet aggregation, endothelial dysfunction, and coronary vasoconstriction [36]. Smoking can also induce autonomic dysfunction. Heavy smoking could impair heart rate variability, heart rate turbulence [37], and HRR [38]. The CARDIA cohort study with 2,730 participants followed for a period of 20 years revealed that current smoking, in addition, to a higher body mass index, larger waist, low education, and fasting glucose level were significant factors independently associated with incident slow HRR [6]. Since smoking is a modifiable risk factor for low HRR and autonomic dysfunction, our study highlights the importance of cessation.

Our findings should be interpreted in light of some limitations. First, previous research indicated that exercise intensity is a key factor in improving HRR. However, exercise, dichotomized based on a cut-off of ≥ 3 days/week with a duration of ≥ 30 min, was not found to be significantly associated with HRR in our study. Future studies should include questions that specifically address the intensity of exercise. Second, our patients were recruited from a single regional hospital and those referred for treadmill exercise test, which might limit the generalizability of our results.

## Conclusions

In this cross-sectional observational study, renal function expressed as either eGFR or CKD stages were significantly and independently associated with one-minute HRR. Our findings suggested that impaired parasympathetic activity recovery after exercise is associated with a decline in renal function.

## Supporting information

S1 Dataset (SAV)
